# Enablers and Barriers to Large-Scale Uptake of Improved Solid Fuel Stoves: A Systematic Review

**DOI:** 10.1289/ehp.1306639

**Published:** 2013-12-03

**Authors:** Eva A. Rehfuess, Elisa Puzzolo, Debbi Stanistreet, Daniel Pope, Nigel G. Bruce

**Affiliations:** 1 Institute for Medical Informatics, Biometry and Epidemiology, Ludwig-Maximilians-University, Munich, Germany; 2 Department of Public Health and Policy, University of Liverpool, Liverpool, United Kingdom

## Abstract

Background: Globally, 2.8 billion people rely on household solid fuels. Reducing the resulting adverse health, environmental, and development consequences will involve transitioning through a mix of clean fuels and improved solid fuel stoves (IS) of demonstrable effectiveness. To date, achieving uptake of IS has presented significant challenges.

Objectives: We performed a systematic review of factors that enable or limit large-scale uptake of IS in low- and middle-income countries.

Methods: We conducted systematic searches through multidisciplinary databases, specialist websites, and consulting experts. The review drew on qualitative, quantitative, and case studies and used standardized methods for screening, data extraction, critical appraisal, and synthesis. We summarized our findings as “factors” relating to one of seven domains—fuel and technology characteristics; household and setting characteristics; knowledge and perceptions; finance, tax, and subsidy aspects; market development; regulation, legislation, and standards; programmatic and policy mechanisms—and also recorded issues that impacted equity.

Results: We identified 31 factors influencing uptake from 57 studies conducted in Asia, Africa, and Latin America. All domains matter. Although factors such as offering technologies that meet household needs and save fuel, user training and support, effective financing, and facilitative government action appear to be critical, none guarantee success: All factors can be influential, depending on context. The nature of available evidence did not permit further prioritization.

Conclusions: Achieving adoption and sustained use of IS at a large scale requires that all factors, spanning household/community and program/societal levels, be assessed and supported by policy. We propose a planning tool that would aid this process and suggest further research to incorporate an evaluation of effectiveness.

Citation: Rehfuess EA, Puzzolo E, Stanistreet D, Pope D, Bruce NG. 2014. Enablers and barriers to large-scale uptake of improved solid fuel stoves: a systematic review. Environ Health Perspect 122:120–130; http://dx.doi.org/10.1289/ehp.1306639

## Introduction

*Household air pollution—a major global health problem*. More than 40% of the world’s population rely for their everyday energy needs on fuels and stove technologies that have changed little since prehistoric times. The transition to modern fuels has been slow in most low-income countries, and because of population growth the number of people using solid fuels (including biomass such as wood, charcoal, dung, or crop residues as well as coal) for cooking has remained at around 2.8 billion since 1990 (Bonjour et al. 2013; Rehfuess et al. 2006). This household energy poverty has multiple consequences for development and, particularly, for health through exposure to very high levels of household air pollution (HAP). Burning solid fuels in open fires or traditional inefficient stoves generates hundreds of pollutants from incomplete combustion, including particulate matter (PM), carbon monoxide, nitrogen oxides, sulfur oxides, polyaromatic and other hydrocarbons, and various organic substances (Naeher et al. 2007). A majority of studies in this field use PM_10_ (PM ≤ 10 µm in aerodynamic diameter) as an indicator pollutant, and average 24-hr concentrations of PM_10_ in solid fuel–using households range from 300 to 3,000 μg/m^3^ (Saksena et al. 2003), greatly exceeding the current World Health Organization (WHO) air quality guidelines for 24-hr and annual mean concentrations of PM_10_ of 50 μg/m^3^ and 20 μg/m^3^, respectively (WHO 2006).

In terms of PM_10_ exposure, HAP can thus be placed somewhere between passive and active smoking and, unsurprisingly, most of the well-known health effects associated with tobacco smoking have also been documented for HAP. Recent systematic reviews show substantially increased risks for acute lower respiratory infections in children (Dherani et al. 2008), low birth weight and stillbirth (Pope et al. 2010), chronic obstructive pulmonary disease (Kurmi et al. 2010), and lung cancer (Hosgood et al. 2011). An increasing number of studies also report a link with cataracts (Pokhrel et al. 2005) and tuberculosis (Sumpter and Chandramohan 2013). Generalized exposure–response functions for combustion-derived PM_2.5_ (≤ 2.5 µm in aerodynamic diameter) (Pope et al. 2011; Smith and Peel 2010) present a strong case for HAP also causing ischemic heart disease and stroke. According to the Global Burden of Disease Project 2010, HAP globally accounted for 3.5 (2.7–4.4) million deaths and 4.3% (3.4–5.3) of disability-adjusted life years in the year 2010 (Lim et al. 2012). Furthermore, 16% of the 3.1 million deaths from outdoor air pollution are also attributable to HAP, due to the impact of household emissions on ambient air (Lim et al. 2012). Based on comparative estimations of the contributions of 67 risk factors in 21 world regions, HAP ranked fourth in terms of global burden (second among women, fifth among men) and accounted for very large fractions of the burden in Sub-Saharan Africa, Oceania, and several Asian regions.

Although the last 30 years have seen a variety of efforts aimed at improving household energy—ranging from small-scale non-governmental organization (NGO)–led projects to the vast Chinese National Improved Stoves Programme (NISP) (Sinton et al. 2004)—most were directed at saving fuel and protecting forests rather than protecting health. In 2012, recognition by the United Nations that energy access is critical for achieving the Millennium Development Goals led to the launch of the Sustainable Energy for All (SEFA) Initiative (SEFA 2013), with ambitious targets for universal access to electricity and modern cooking energy systems by 2030. Complementing and contributing to this global initiative are a range of national, regional, and international strategies, in particular the Global Alliance for Clean Cookstoves (2013), with its target to establish a sustainable global market for clean stoves and fuels.

*An important role for improved solid fuel stoves*. SEFA and other strategies for moving the world toward the 2030 targets envisage a mix of interventions. In favorable settings, where biomass fuels are already purchased and/or households possess the necessary economic means, a relatively rapid shift to clean fuels is feasible. At the same time, households unable to afford and/or access modern fuels in the short- to medium-term must have access to solid fuel stoves that are as clean and safe as possible. The International Energy Agency (IEA) has proposed distinct regional scenarios based on a combination of liquefied petroleum gas (LPG), biogas, and low-emission solid fuel stoves in which, of a total of 129 million people gaining access to all three types of modern energy worldwide, 59 million (46%) per year should gain access to improved solid fuel stoves (IS) (IEA 2011). Whatever the mix of fuels and technologies ultimately adopted by households over the next 20 years, improved solid fuel stoves will continue to play a very important part.

Over the last three decades, the term “improved stove” has been variably applied to describe stove models optimized for fuel efficiency or designed to minimize emissions. Consequently, their effectiveness in reducing health-damaging emissions has been highly variable (Bruce et al. 2006). Evidence is, however, emerging of the potential of IS to deliver at least some of the health benefits promised by the mainly observational epidemiological evidence on risks of exposure. The RESPIRE trial (Randomized Exposure Study of Pollution Indoors and Respiratory Effects) found that the *plancha* chimney stove used widely across Guatemala and other Latin American countries reduced kitchen pollution concentrations by 90% and children’s and women’s exposures by 60% and 50%, respectively (Smith et al. 2011). For children ≤ 18 months of age, this was associated with a 33% (95% CI: 2, 55%) reduction in severe pneumonia incidence (Smith et al. 2011). Likewise, three cohort studies investigating the impacts of chimney stoves disseminated as part of the Chinese NISP reported 25–50% reductions in risk of chronic obstructive pulmonary disease, lung cancer, and adult pneumonia mortality, although these studies lacked exposure measurement (Chapman et al. 2005; Lan et al. 2002; Shen et al. 2009). Economic modeling suggests that IS use can be a cost-effective means of reducing the HAP-attributable disease burden (Mehta and Shahpar 2004) and show highly favorable cost–benefit ratios when examined from a societal perspective (Hutton et al. 2007; Malla et al. 2011). There are, however, recent warnings that stoves that are not well suited to household needs may fail to deliver health benefits (Hanna et al. 2012).

Despite the widespread perception that achieving uptake of IS at scale presents significant policy and programmatic challenges, this issue has received relatively little attention. Understanding factors that make projects and programs succeed or fail will be critically important to achieve the “quantum leap” (Rehfuess 2006) required for sustainable adoption of IS by hundreds of millions of households. The aim of this systematic review was to contribute to filling this evidence gap by identifying factors that enable or limit household uptake of improved solid fuel stoves in low- and middle-income countries. Very few improved stove initiatives to date have demonstrated health and broader benefits at scale and in a sustainable way. Consequently, a systematic review of past experience is likely to provide relevant cues to significant obstacles and facilitators but is unlikely to generate a reliable and easily replicable “recipe” to guarantee the success of future initiatives.

## Methods

*Scope of systematic review*. This review forms part of a broader systematic review concerned with enablers of and barriers to large-scale uptake of a range of household energy technologies in the context of projects, programs, or other relevant initiatives undertaken at any scale; findings related to clean fuels will be reported elsewhere. In principle, IS encompass a wide variety of designs and technologies, ranging from user-built stoves made of locally available materials to mass-produced advanced combustion stoves. These differ greatly in their suitability for different cooking practices and other household energy requirements as well as in their emissions of PM, carbon monoxide, and other health-damaging pollutants and in their fuel efficiency and safety. Although the effectiveness of the interventions is of fundamental importance, this review was principally concerned with uptake and, accordingly, we considered all IS types.

**Figure 1:**
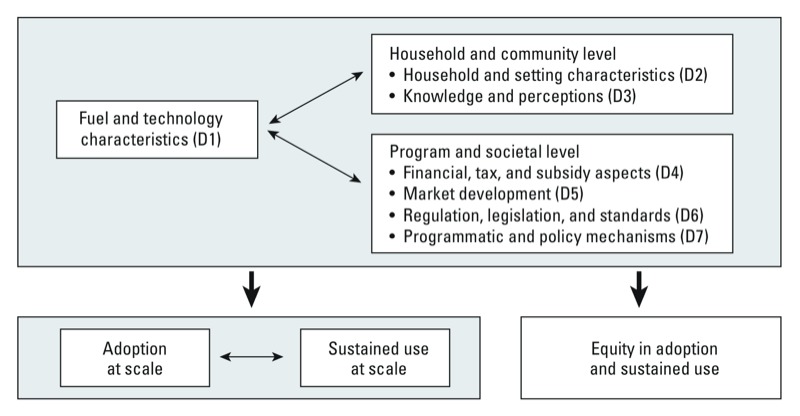
Framework of domains for the factors enabling or limiting uptake of cleaner cooking technologies. This framework illustrates how seven domains (D)—one relating to the characteristics of the intervention, two operating at the household/community level, and four operating at the program/societal level—affect uptake of IS. Uptake at scale comprises short-term adoption as well as longer-term sustained use and may take place in equitable or less equitable ways. Factors within the seven domains may enable or limit one or several aspects of adoption, sustained use, and equity.

As a means of structuring the review, a comprehensive framework of factors influencing uptake of cleaner cooking technologies was developed, drawing on previous work (Bruce et al. 2006; World Bank 2011). This framework encompasses seven domains. The framework highlights the central role of fuel and technology characteristics, and shows how two other domains—characteristics of households and settings; knowledge and perceptions—primarily operate at the household and community level. The remaining four domains—financial, tax, and subsidy aspects; market development; regulation, legislation, and standards; programmatic and policy mechanisms—primarily operate at the program and societal level (Figure 1). Enabling or limiting factors affecting short-term adoption may differ from those affecting longer-term sustained use. In addition, uptake may occur equally or unequally across population groups differing by socioeconomic status and urban–rural location, and it is likely to be influenced by gender-related factors. For this review, which draws on and further develops concepts advanced in the literature (Pine et al. 2011; Ruiz-Mercado et al. 2011), IS adoption is defined to include both acquisition (stoves are purchased or installed without any reference to their later use) and initial adoption (use is assessed < 1 year from acquisition). Sustained use, on the other hand, comprises both medium-term (assessed 1–2 years after acquisition) and long-term sustained use (reflecting longer time periods).

This systematic review was registered with the Evidence for Policy and Practice Information and Co-ordinating Centre (EPPI-Centre) at the University of London, and a detailed, peer-reviewed protocol is available (Puzzolo et al. 2011).

*Search strategy*. We adopted an exhaustive search strategy comprising

Systematic searches in 27 peer-reviewed databases across multiple disciplines [including MEDLINE (http://www.ncbi.nlm.nih.gov/pubmed/), EMBASE (http://www.elsevier.com/online-tools/embase), and social science databases]
Gray literature searches of 14 portals of key stakeholder organizations, such as the HEDON Household Energy Network (http://www.hedon.info/) and the Partnership for Clean Indoor Air (http://www.pciaonline.org/), complemented by searches through Google (http://www.google.com/) and Google Scholar (http://scholar.google.com/)
Handsearches of the references of included studies
Expert consultations.

Full details are available in the protocol (Puzzolo et al. 2011).

For bibliographic databases, comprehensive search terms representing “interventions”

(*stove OR (cook* AND technol*) OR (cook* AND fuel) OR LPG OR “LP gas” OR “liquid petroleum gas” OR “liquefied petroleum gas” OR “liquified petroleum gas” OR chulha OR chulla OR challah OR chula)

were combined with search terms representing “uptake”

(adopt* OR accept* OR deliver* OR dissemin* OR implement* OR scale OR “scal* up” OR “roll* out” OR “tak* up” OR uptake)

using the Boolean operator AND. Piloting of the terms was carried out; small modifications to meet the needs of specific databases were made whenever necessary. We conducted searches of the period 1980–July 2011, using English terms, and we screened publications in English, Spanish, Portuguese, French, German, and Italian.

*Inclusion/exclusion criteria*. To be eligible for inclusion, studies had to relate to direct experience with IS and to provide empirical information on factors influencing adoption or sustained use. Projects and programs were considered only if they targeted households rather than public or commercial settings, with restriction to urban and rural areas of low- and middle-income countries defined according to World Bank regions (2014). Studies undertaken in refugee camps were excluded because of limited generalizability.

Recognizing that uptake of IS is influenced by factors operating at all levels in society (Figure 1), we set up this review to encompass three types of studies:

Qualitative studies, conducted as part of an intervention study or stand-alone
Quantitative epidemiological studies
Case studies drawing on multiple sources of information to provide a broad evaluation of a specific project, program, or policy.

To qualify for inclusion, case studies had to *a*) rely on at least one source of empirical information; *b*) report information on sampling, data collection, and/or analysis; and *c*) provide some analysis of the factors influencing success/failure of IS uptake.

To identify eligible studies, titles and abstracts were screened by one author, with independent random checks of included (10%) and excluded (10%) abstracts. Full text articles for initial consideration were independently screened by two or more authors. All decisions were documented using the software EPPI Reviewer 4 (http://eppi.ioe.ac.uk/cms/Default.aspx?alias=eppi.ioe.ac.uk/cms/er4).

*Data extraction and quality appraisal*. Data extraction for included studies was conducted by one author using standardized forms, and checked by two authors during synthesis. Key findings and characteristics of studies were reported in summary tables. Qualitative studies were appraised for quality using established criteria related to reliability and validity of findings and the reflection of participant perspectives (Harden et al. 2009; Appendix 2.7 in Puzzolo et al. 2013). Quantitative studies were appraised for quality using Liverpool Quality Assessment Tools (Appendix 2.8 in Puzzolo et al. 2013) to assess design-specific sources of potential bias and confounding. The quality of case studies was examined by adapting published criteria for case studies (Atkins and Sampson 2002; Appendix 2.9 in Puzzolo et al. 2013), with a particular emphasis on distinguishing between empirical analysis and subjective author interpretation. Quality appraisal was independently conducted by two authors; any discrepancies were resolved through discussion between two or more authors. Results of quality appraisal were categorized as strong, moderate, or weak. However, quality appraisal across study designs is not directly comparable.

*Evidence synthesis*. Synthesis of extracted studies was carried out in two stages. In the first stage, synthesis was conducted separately for the three types of studies, referencing detailed findings so they would remain traceable to individual studies. For qualitative studies, we used thematic synthesis, as developed and applied by Thomas and Harden (2008). Line-by-line coding of the full text was followed by generation of descriptive themes for each study; these were compared across studies and synthesized under the seven framework domains and for equity in tabular and narrative form (Puzzolo et al. 2011). For quantitative and case studies, quantitative and/or descriptive findings in each study were extracted onto data extraction forms. Subsequently, findings were compiled into two tables—one for all quantitative studies, and one for all case studies—and organized as specific enablers or barriers under the seven framework domains and equity. For each domain, related enablers and barriers were grouped and relevant headings were assigned. Each of these headings was thereafter referred to as a “factor.” Specific findings for each factor were also described in narrative form.

In the second stage, synthesis of evidence relating to each factor was conducted by drawing on the information from all three study types. We found that preserving a distinction between barriers and enablers was not meaningful: It became apparent that most factors operate along a spectrum, where they enable uptake when present or satisfactory, or limit uptake when absent or unsatisfactory. We assessed the strength of evidence supporting each factor by consistency across study types and countries and settings, as well as by number and quality of studies. We also conducted a graphical sensitivity analysis, based on moderate and strong studies.

## Results

*Description of included studies*. Of 6,690 unique records identified, 57 studies met the inclusion criteria (14 qualitative, 16 quantitative, and 27 case studies) (see Supplemental Material, Figure S1). Notably, Bangladesh (*n* = 8), India (*n* = 17), Kenya (*n* = 5), and Mexico (*n* = 6) contributed a large number of studies. Of the 57 studies, 31 were undertaken in rural areas and 11 in urban areas, whereas 15 covered both settings. Table 1 shows the basic study characteristics: 35 studies were concerned with adoption and 13 studies with sustained use, whereas 9 studies assessed elements of both adoption and sustained use. Detailed study characteristics are available in Supplemental Material, Table S1, which shows a range of stove types as determined by production materials, main fuel use, number of potholes, and ventilation. The majority of studies were concerned with stoves produced by local artisans, and 7 studies contributed findings for more standardized stove production.

**Table 1:** Basic characteristics of included studies.

Type of evidence/study ID	Reference^*a*^	Country	Setting	Adoption	Sustained use
(A) Qualitative studies
A1	Anderson 2007	India	Rural		✔
A2	Chowdhury et al. 2011	Bangladesh	Rural	✔	✔
A3	Christoff 2010	Mexico	Rural	✔
A4	Gordon et al. 2007	Mongolia	Urban	✔
A5	Jagoe et al.^*b*^	India	Rural	✔
A6	Jagoe et al.^*c*^	India	Rural	✔
A7	Pandey 1989	Nepal	Rural	✔	✔
A8	Person et al. 2012	Kenya	Rural	✔
A9	Sesan 2012	Kenya	Urban^*d*^	✔
A10	Simon 2007	India	Rural	✔	✔
A11	Sovacool and Drupady 2011	Bangladesh	Rural/urban	✔	✔
A12	Troncoso et al. 2007	Mexico	Rural	✔
A13	Troncoso et al. 2011	Mexico	Rural	✔
A14	Velasco 2008	Mexico	Rural	✔
(B) Quantitative studies
B1	Agurto-Adrianzen 2009	Peru	Rural	✔
B2	Bensch and Peters 2011	Senegal	Urban	✔
B3	Damte and Koch 2011	Ethiopia	Urban		✔
B4	El Tayeb Muneer and Mukhtar Mohammed 2003	Sudan	Rural/urban	✔
B5	George and Yadla 1995	India	Rural	✔
B6	Inayatullah 2011	Pakistan	Rural	✔
B7	Jagoe et al.^*b*^	India	Rural	✔
B8	Jagoe et al.^*c*^	India	Rural	✔
B9	Levine and Cotterman 2012	Uganda	Urban	✔
B10	Miller and Mobarak 2011	Bangladesh	Rural	✔
B11	Mwangi 1992	Kenya	Rural	✔
B12	Pandey and Yadama 1992	Nepal	Rural	✔
B13	Pine et al. 2011	Mexico	Rural	✔
B14	Pushpa 2011	India	Rural	✔
B15	Silk et al. 2012	Kenya	Rural	✔
B16	Wallmo and Jacobson 1998	Uganda	Rural	✔
(C) Case studies
C1	Amarasekera 1989	Sri Lanka	Rural/urban	✔	
C2	Barnes et al. 2012a	India, Western Maharashtra	Rural/urban		✔
C3	Barnes et al. 2012b	India, Haryana	Rural/urban		✔
C4	Barnes et al. 2012c	India, Karnataka	Rural/urban		✔
C5	Barnes et al. 2012d	India, Gujarat	Rural/urban		✔
C6	Barnes et al. 2012e	India, Andhra Pradesh	Rural/urban		✔
C7	Barnes et al. 2012f	India, West Bengal	Rural/urban		✔
C8	GERES 2009	Cambodia	Urban	✔	✔
C9	Kürschner et al. 2009	Bangladesh	Rural/urban	✔	✔
C10	Masera et al. 2005	Mexico	Rural	✔
C11	Mounkaila 1989	Niger	Urban	✔
C12	Namuye 1989	Kenya	Urban	✔
C13	Osei 2010	Ghana	Rural/urban	✔
C14	Sawadogo 1989	Burkina Faso	Urban	✔
C15	Shastri et al. 2002	India	Rural		✔
C16	Shrimali et al. 2011	India	Rural/urban		✔
C17	Simon 2010	India	Rural	✔	✔
C18	Sinton et al. 2004	China	Rural/urban		✔
C19	Sudjarwo et al. 1989	Indonesia	Rural	✔	✔
C20	USAID/Winrock 2008	Peru	Rural		✔
C21	USAID/Winrock 2009	Bangladesh	Urban	✔
C22	World Bank 2004a	Guatemala	Rural	✔
C23	World Bank 2004b	Guatemala	Rural	✔
C24	World Bank 2004c	Guatemala	Rural	✔
C25	World Bank 2010a	Bangladesh	Rural/urban		✔
C26	World Bank 2010b	Bangladesh	Rural/urban		✔
C27	World Bank 2010c	Bangladesh	Urban	✔
Abbreviations: GERES, Groupe Energies Renouvelables, Environnement et Solidarités; ID, identification; USAID, U.S. Agency for International Development. ^***a***^For more information, see Supplemental Material, Table S1. ^***b***^Jagoe K, Bromley H, Chengappa C, Bruce NG, unpublished data. ^***b***^Jagoe K, Bromley H, Dutta K, Bruce NG, unpublished data. ^***d***^Conducted in a peri-urban setting.					

Qualitative studies comprised interviews, focus groups, and ethnographic studies. A poor description of the theoretical approach to analysis and of strategies employed to increase the validity of findings, as well as limited distinctions between findings emerging from the research and subjective author interpretations, were common problems. Of the 14 qualitative studies, 5, 8, and 1 study were categorized as strong, moderate, and weak, respectively. The 16 quantitative studies comprised controlled trials, cross-sectional surveys, and economic analyses. Of the 16 quantitative studies, 4, 7, and 5 studies were classified as strong, moderate, and weak, respectively, with the main areas of weakness being poor sampling methods and relatively simple descriptive analyses. The 27 case studies varied greatly in terms of the combination of direct empirical findings (e.g., cross-sectional surveys, focus groups, interviews), reference to publicly available statistics, and more subjective program experience or opinion. Of the 27 case studies, 10, 12, and 5 were considered to be strong, moderate, and weak, respectively.

**Figure 2:**
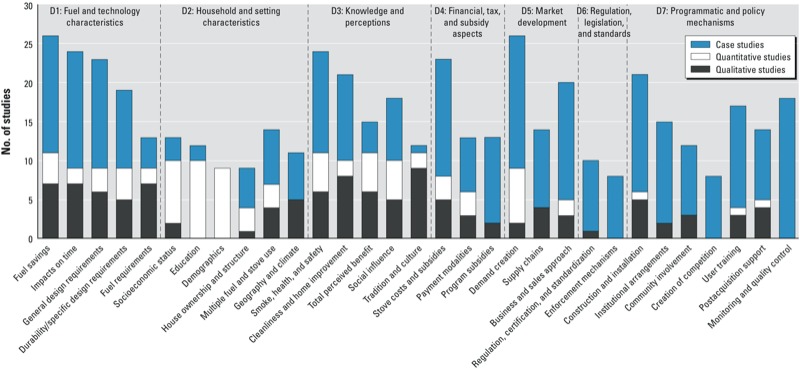
Factors influencing uptake of improved solid fuel stoves across seven domains (D).

Next, we present the second stage of evidence synthesis [findings from individual studies and from the first stage of synthesis by study type are available elsewhere (Puzzolo et al. 2013)]. Table 2 lists factors under each domain (D1 through D7) supported by references [i.e., study identification (ID)], recording study types and the countries where the evidence was collected. The sections below summarize findings under the relevant domains, as well as for equity, and provide cross-links to factors in Table 2. Figure 2 graphically displays all 31 factors and the number of quantitative, qualitative, and case studies supporting them. Supplemental Material, Figure S2, shows the results of the graphical sensitivity analysis based on moderate and strong studies, with the findings regarding different factors being largely comparable to the main analysis.

**Table 2:** Enabling and limiting factors for uptake of improved stoves.

Domain/factor	Type of evidence	Country, study ID,^*a*^ and setting^*b*^
D1. Fuel and technology characteristics
Fuel savings	A, B, C	Bangladesh^A2,B10,C9,C27^, Burkina Faso^C14^, Cambodia^C8^, Guatemala^C22^, India^A1,A5,A10,B7,C2–C7,C16^, Kenya^A8,C12^, Mongolia^A4^, Mexico^A12^, Nepal^B12^, Niger^C11^, Sri Lanka^C1^, Uganda^B16^
Impacts on time	A, B, C	Bangladesh^A11,C9^, Burkina Faso^C14^, Cambodia^C8^, Guatemala^C22,C24^, India^A1,A6,C2–C7,C16^, Indonesia^C19^, Kenya^C12^, Mexico^A3,A12,A14^, Nepal^A7,B12^, Sri Lanka^C1^, Uganda^B16^
General design requirements	A, B, C	Bangladesh^C21^, Cambodia^C8^, China^C18^, Guatemala^C22–C24^, India^A1,A5,A6,B8,C2–C7,C17^, Indonesia^C19^, Mexico^A3,A12,A13,B13^, Nepal^B12^, Uganda^B16^
Durability and other specific design requirements	A, B, C	Bangladesh^C9^, Burkina Faso^C14^, Guatemala^C22^, India^A1,A6,B8,C7,C17^, Indonesia^C19^, Kenya^C12^, Mexico^A3,A12,C10^, Nepal^B12^, Niger^C11^, Sri Lanka^C1^, Uganda^B9,B16^
Fuel requirements	A, B, C	Bangladesh^A2,A11^, Guatemala^C24^, India^A1,C7,C17^, Indonesia^C19^, Mexico^A3,A12,A13^, Nepal^A7,B12^, Uganda^B16^
D2. Household and setting characteristics
Socioeconomic status	A, B, C	Burkina Faso^C14^, Ethiopia^B3^, India^B14,C16^, Indonesia^C19^, Kenya^A8,A9,B11,B15^, Pakistan^B6^, Peru^B1^, Senegal^B2^, Sudan^B4^
Education	B, C	Bangladesh^B10^, Ethiopia^B3^, India^B5,B14^, Indonesia^C19^, Kenya^B15^, Mexico^B13^, Pakistan^B6^, Peru ^B1^, Sri Lanka^C1^, Senegal^B2^, Sudan^B4^
Demographics	B	Ethiopia^B3^, India^B14^, Kenya^B11,B15^, Mexico^B13^, Pakistan^B6^, Peru^B1^, Sudan^B4^, Uganda^B9^
House ownership and structure	A, B, C	Ethiopia^B3^, Kenya^A9^, India^C3–C5^, Mexico^B13,C10^, Peru^C20^, Uganda^B16^
Multiple fuel and stove use	A, B, C	Cambodia^C8^, India^A10,B7,C4,C5,C16^, Indonesia^C19^, Mexico^A12–A14,B13,C10^, Pakistan^B6^, Sri Lanka^C1^
Geography and climate	A, C	Bangladesh^A11,C9^, Cambodia^C8^, Guatemala^C23^, India^A1,C3,C16^, Kenya^A8^, Mexico ^A12^, Mongolia^A4^, Niger^C11^
D3. Knowledge and perceptions
Smoke, health, and safety	A, B, C	Bangladesh^A11,B10^, Cambodia^C8^, Guatemala^C23^, India^A1,B8,C2–C7,C16^, Indonesia^C19^, Kenya^A9,C12^, Mexico^A3,A14,B13,C10^, Mongolia^A4^, Nepal^B12^, Niger^C11^, Uganda^B16^
Cleanliness and home improvement	A, B, C	Guatemala^C23,C24,^ India^A5,B8,C2–C7^, Kenya^A8,A9,C12^, Mexico^A3,A12,A14,C10^, Mongolia^A4^, Nepal^A7^, Niger^C11^, Uganda^B16^
Total perceived benefit	A, B, C	Bangladesh^C21^, India^A1,A6,B8,B14,C4,C6^, Kenya^A8,A9^, Mexico^A12^, Nepal^A7,B12^, Niger^C11^, Sudan^B4^, Uganda^B16^
Social influence	A, B, C	Bangladesh^B10^, India^C2–C4^, Indonesia^C19^, Kenya^A8,C12^, Mexico^A3,A12,A14,B13,C10^, Nepal^A7,B12^, Niger^C11^, Peru^B1,C20^, Uganda^B16^
Tradition and culture	A, B, C	Bangladesh^A11^, India^A1,A5,A6,C5^, Kenya^A8^, Mexico^A3,A12,A13^, Nepal^A7,B12^, Uganda^B16^
D4. Financial, tax, and subsidy aspects
Stove costs and subsidies	A, B, C	Bangladesh^B10,C26,C27^, Guatemala^C22–C24^, India^A1,A10,C2,C7,C17^, Indonesia^C19^, Kenya^A8,A9,C12^, Mongolia^A4^, Niger^C11^, Uganda^B9,B16^
Payment modalities	A, B, C	Bangladesh^B10,C21,C26^, Ghana^C13^, Guatemala^C22,^ India^A5,A6,B8,C16^, Kenya^A9^, Mexico^C10^, Peru^C20^, Uganda^B9^
Program subsidies	A, C	Bangladesh^C9,C21,C25^, China^C18^, Ghana^C13^, Guatemala^C22–C24,^ India^C5,C6,C16^, Kenya^A9^, Mexico^A13^
D5. Market development
Demand creation	A, B, C	Bangladesh^B10, C9,C21,C25,C27^, Burkina Faso^C14^, Ethiopia^B3^, Guatemala^C22–C24,^ India^A10,C3,C16–C17^, Indonesia^C19^, Kenya^A8,B11,B15,C12^, Mexico^C10^, Niger^C11^, Peru^C20^, Sudan^B4^, Uganda^B9,B16^
Supply chains	A, C	Bangladesh^A11,C21,C25^, Ghana^C13^, Guatemala^C22,C23^, India^A1,A10,C6,C16^, Indonesia^C19^, Kenya^A8^, Niger^C11^, Sri Lanka^C1^
Business and sales approach	A, B, C	Bangladesh^A11,C9,C21^, Cambodia^C8^, Ghana^C13^, Guatemala^C22,C23^, India^A10,C3–C6,C16,C17^, Indonesia^C19^, Kenya^A8,B15^, Mexico^C10^, Uganda^B9^
D6. Regulation, legislation, and standards
Regulation, certification, and standardization	A, C	Cambodia^C8^, China^C18^, Guatemala^C22^, Kenya^A8,C12^, India^C4,C6,C7,C16^, Niger^C11^
Enforcement mechanisms	C	Cambodia^C8^, China^C18^, India^C2,C3,C5,C7,C16,C17^
D7. Programmatic and policy mechanisms
Construction and installation	A, B, C	Bangladesh^A2,C26,C9^, Cambodia^C8^, China^C18^, Guatemala^C22–C24,^ India^A6,B5,C2–C7^, Mexico^A3,A12^, Nepal^A7^, Peru^C20^, Sri Lanka^C1^
Institutional arrangements	A, C	Bangladesh^A11,C25^, China^C18^, Guatemala^C23,C24,^ India^C2–C7,C16,C17,^, Kenya^A9^, Sri Lanka^C1^
Community involvement	A, C	Bangladesh^C21,C26^, Guatemala^C22^, India^A10,C2–C6^, Kenya^A9^, Mexico^A13,C10^
Creation of competition	C	Cambodia^C8^, China^C18^, India^C2–C5,C7^, Peru^C20^
User training	A, B, C	Bangladesh^A11,C9,C25,C26^, Guatemala^C22,C23^, India^B5,C2–C5,C7,C16^, Indonesia^C19^, Mexico^A3,A12,C10^
Post­acquisition support	A, B, C	Bangladesh^A11,C9,C25,C26^, India^A10,B5,C2–C4,C6,C7,C16^, Mexico^A3,A13^
Monitoring and quality control	C	Bangladesh^C9,C21,C25,C27^, Cambodia^C8^, Guatemala^C22–C24^, India^C2–C7,C16^, Indonesia^C19^, Mexico^C10^, Niger^C11^
Abbreviations: A, Qualitative studies; B, quantitative studies; C, case studies. ^***a***^Study IDs are listed in Table 1. ^***b***^All factors are supported by findings in rural as well as urban settings.		

*Domain 1: Fuel and technology characteristics*. Many of the studies confirmed the fundamental requirement that IS are designed to meet user needs in preparing local dishes with traditional cooking utensils and available fuels (general design requirements). Where relevant, stove designs must also meet other household energy needs such as seasonal space heating. Failure to effectively address these issues almost guarantees that the improved stove will not be adopted and used long-term or that it will be used for some but not the majority of purposes. Household requirements are rarely met in a “one-size-fits-all” fashion, emphasizing the importance of incorporating user requirements in research and development and of offering a choice of high-quality designs.

Even if the stove is well-designed to meet local needs, its use will decline if durability is poor and chimneys (where used) break or become blocked quickly (durability and other specific design requirements). Design and durability also affect the requirements for, and costs of, cleaning and maintenance, which can be a disincentive if high. Many programs report that a range of specific design problems (e.g., small size of stove entrance) lead to stove modifications by users limiting stove effectiveness or prompting the reversion to traditional stoves. On the positive side, stoves with a modern, attractive appearance can be highly valued.

Fuel savings, whether perceived or measured, are widely reported as an important incentive. Fuel savings comprise savings in fuel collection time (and associated injuries and threats) and/or household expenditure when fuel is bought. Some IS, however, are more restrictive in the type and size of fuel required, which may be a barrier to adoption and use (fuel requirements). The other major area of savings related to stove performance is time, which, in addition to time saved by less fuel collection, may be achieved through more efficient cooking due to greater heat transfer efficiency or parallel cooking on multiple potholes (impacts on time). However, the requirement for fuel processing (e.g., chopping wood into smaller pieces) or regular stove and chimney cleaning may add work. Time savings are reported to be used for other household work or income generation, but the attached value varies between settings. Notably, several studies found that poor rural communities, where fuel and labor are both abundant, do not consider the opportunity costs of time spent on cooking or fuel collection to be important.

*Domain 2: Household and setting characteristics*. This domain comprises the socioeconomic, demographic, structural, and geographical contexts of households, which interact to affect adoption and sustained use. Higher socioeconomic status, related to income, household assets, or expenditure, is widely found to enable adoption. Similarly, greater education (years of schooling or educational attainment) among women and men, increases uptake. Somewhat less consistent results emerge for demographic characteristics, notably with respect to sex and age of the head of household (demographics). Larger family size appears to act as a barrier to adoption, possibly due to the low value assigned to time and labor used to collect firewood and/or the need to cook for more people. On the other hand, house ownership is an enabler, which is likely to partially reflect socioeconomic status, but also willingness to invest in home improvements (house ownership and structure). In fact, the lack of a permanent home or kitchen, as well as space limitations, can be impediments to purchasing a built-in stove.

A generic issue at the household level, emerging from many countries and settings and having far-reaching implications, is the phenomenon of habitual “fuel/stove stacking.” This describes multiple fuel and stove use, which may include a variety of solid fuels, an improved stove used alongside a preexisting (set of) traditional stove(s), or solid fuels used in combination with LPG or kerosene. Existing fuel/stove stacking enables uptake of an additional technology, but it also acts as a barrier to exclusive use of IS. Households that purchase rather than collect solid fuels are more likely to adopt, reflecting the greater perceived value of monetary savings compared to time savings.

Geography and climate are also important influences on uptake. Urban households are generally more willing to adopt, whereas those in disaster-prone areas may be less willing or less able. Those living at higher altitude and in other cold settings require interventions that also provide warmth. In some settings, the IS must also take seasonal demands into account. For example, stove portability is valued where households switch between outdoor cooking during the dry season and indoor cooking during the rainy season.

*Domain 3: Knowledge and perceptions*. This domain addresses the perceptions and expectations of users, mostly women, regarding the impact of IS on their daily lives. A prerequisite for adoption and sustained IS use is that users should be able to prepare their local dishes to the same taste, using established cooking utensils, especially in view of resistance to changing traditional practices (tradition and culture). The ability to cook for larger gatherings is also important in many settings.

Fewer adverse health effects, especially those directly perceived to be smoke-related, and a reduction in risk of burn injuries and house fires emerge as enablers for adoption and sustained use (smoke, health and safety). By contrast, the perception that smoke protects against insects concerned households in two Indian studies. Likewise, cleaner homes and cooking vessels are appreciated by users of IS. Other reported benefits enabling adoption include emitted warmth, the family eating together, and children being able to study/play indoors (cleanliness and home improvement).

Where the advantages of IS outweigh those associated with traditional practices, households perceive the investment as a good value for the money. Where the improved stove does not meet expectations and there are competing household priorities, such as food security, willingness to pay is limited. Beyond considerations of total perceived benefit to the household, social networks and opinion leaders influence adoption in positive and negative ways (social influence). A bad experience with the technology is especially destructive in this regard. IS can be an aspirational choice when seen to have aesthetic appeal and associated status gain.

*Domain 4: Financial, tax, and subsidy aspects*. Findings for this domain are derived from government-led, NGO-led, and market-based dissemination approaches. Whatever the approach, the cost of high-quality IS is an important barrier to adoption and/or repurchase, which may be overcome through government- or market-led economies of scale or stove subsidies (stove costs and subsidies). Much of the evidence on stove subsidies comes from India. Subsidies toward the stove or its component parts enable initial adoption, with several studies emphasizing that the poorest households would not have gained access to IS without them. Large subsidies can, however, devalue IS, limiting maintenance efforts and longer-term use and repurchase by households and, through subsidy expectations into the future, program sustainability. Insights relating to flexible versus fixed pricing policies are mixed, although a multi-tier scheme (prices scaled for stove model) was reported to be effective in some Indian and Bangladeshi settings.

Liquidity constraints, especially among poorer populations, limit uptake. Although payments in installments, price incentives or other promotional offers, and consumer finance through microcredit/loans (offered through local companies or community lending schemes) are mechanisms to overcome this barrier, their relevance and success varied according to stove price and target population (payment modalities). For example, microcredit was successfully employed to reach urban and rural poor households in Bangladesh, although in some settings short payback periods and high interest rates deterred those eligible from applying for microcredit.

Importantly, independent of the underlying ideology or programmatic approach, most programs will benefit from some degree of government support (program subsidies). Direct/indirect government financial support (e.g., grants, loans, tax incentives) toward improved stove programs is a major enabler of uptake, especially in relation to adequate up-front entrepreneurial capital for stove business development. Financial incentives for stove construction and maintenance and support toward research and development and raising awareness are also important. Any dependence on national or international external support and supplies should be carefully evaluated for sustainability.

*Domain 5: Market development*. Creating demand through appropriate and, potentially, setting-specific strategies is important for stove uptake (demand creation). Modes of demand creation comprise general awareness-raising activities about the benefits of IS (e.g., through media campaigns) and personal contact through women’s organizations or company representatives. Product demonstrations and “word-of-mouth” advertising appear to be the most important general drivers of adoption. A demand-driven approach facilitates long-term adoption and use, whereas coercive approaches based on deliberate misinformation or false promises are likely to favor rejection of the technology despite initial uptake. Demand can be met only if those raw materials, stove parts, or complete stoves not available locally are accessible to users through well-managed supply chains. Supply chains may be newly established or make use of existing production and dissemination networks. Road infrastructure has an impact on distribution and availability, including prices, and may be a precondition for supply of stoves that are not locally produced.

Both government-led and market-based programmatic approaches ultimately rely on functional, self-sustaining businesses to produce, disseminate, and maintain IS in order to be successful (business and sales approach). Findings with respect to the success of mass production versus artisanal production are inconclusive, in part because each choice is often intrinsically linked to the overall programmatic approach. The challenge to sustain income is an important issue for IS businesses. Despite the potentially large unmet demand, the experience of many Indian stove companies suggests that a relatively poor market segment and the seasonality of stove production result in modest returns. Approaches adopted to ensure sustained income among small- and larger-scale producers include *a*) combining sales through a government program with sales on the open market; *b*) cross-subsidizing sales to households through sales to institutional customers; *c*) specializing in the production of stove parts; *d*) pursuing indirect sales via outlets or direct sales via manufacturers; *e*) exploring opportunities for the joint sale of two or more products; or *f*) ensuring an independent second source of income. Overall, an entrepreneurial mode and appropriate business skills emerge as keys to success and financial viability; however, the lack of interest in providing after-sales services may be a barrier to sustained use.

*Domain 6: Regulation, legislation, and standards*. Relatively few studies report on the role of this domain, but the clear message is that standards and their enforcement are critical for large-scale promotion of high-quality IS. Although the limited findings did not suggest a strong enabling or limiting role for state control on fuel and raw material pricing, subsidies for kerosene or LPG can create market distortions, acting against IS uptake. Certification of stove or stove component manufacturers by a standards agency or a network of producers is a means of ensuring adherence to design specifications for fuel efficiency and emissions (regulation, certification, and standardization). Certification must be enforced through mechanisms such as the procurement of materials from designated suppliers, the exclusive use of accredited manufacturers, and penalties to revoke accreditation in the case of noncompliance with standards (enforcement mechanisms).

*Domain 7: Programmatic and policy mechanisms*. This domain addresses interactions between different stakeholders and specific aspects of program planning and implementation, monitoring, and quality control.

Coordination and regular interaction between stakeholders—be they government agencies, NGOs, private sector entities, or targeted households and communities—and careful program management with good feedback systems are enablers of uptake (institutional arrangements). Where appropriate, synergies may be achieved through integration of IS programs with broader rural development programs. The Indian experience suggests that state- versus market-based programs show distinct strengths and weaknesses. The former tend to create dependence on public support and control technology innovation but minimize opportunities for corruption, whereas the latter enable technology innovation by local partners but may be liable to favoritism. Independent of programmatic approach, government has a key role in ensuring links to relevant mainstream policy, research and development for stove technologies, health campaigns, and financial oversight. Short-term, target-bound programs—frequently related to strict funding schemes—often fail to achieve sustainable change. Community involvement, from identification of suitable stove designs to stove distribution, creates a greater sense of ownership. Fostering women’s engagement is particularly important.

Several programs have successfully employed competition and reward schemes—between women or households (Bangladesh, India), producers or implementers (Cambodia, India), villages and/or counties (China, India)—to encourage uptake and sustained use (creation of competition). Targeting of market segments has also been successfully employed: In selected Indian states, program preference for villages or districts expressing high demand for improved stoves has led to high coverage rates in these selected locations; and in the Chinese NISP’s competitive focus on wealthier counties, local co-funding was critical for rapid program uptake at scale.

All stages, from choice of raw materials for stove construction to postacquisition support, need to be considered in program planning and implementation. Adherence to design specifications and high-quality construction and installation are important. Faulty construction and/or installation of IS and, where applicable, chimneys lead to reduced functionality and durability. Appropriate training for stove builders in construction and entrepreneurship and establishing the stove business as a profession, rather than a casual job, are thus critical enablers of uptake. Insufficient user training on stove (and chimney) use, cleaning, and maintenance negatively affect functionality and sustained use, potentially leading to frustration (user training). Hands-on training tends to be more effective than the provision of an instruction manual. Lack of or poor-quality postacquisition support is an important barrier to sustained use, as identified across many studies and settings. Consequently, there is a stated need for a formal policy, for example, combining free after-sales services during a warranty period with subsequent services for payment.

Equally, a lack of appropriate monitoring and quality control mechanisms is widely reported as a barrier. Sufficient technical personnel and adequate financial resources for monitoring at preconstruction, construction, and installation phases as well as post-construction should be ensured. Responsiveness at all levels to concerns expressed by users, producers, and implementers is critical.

*Equity in adoption and sustained use*. Equity considerations are critical in scaling-up improved stove dissemination to those of the lowest socioeconomic status, living in rural or remote areas, and women experiencing the greatest health risks associated with traditional household energy practices. Yet, these groups are most often also the least able to access or afford IS.

Programs with an explicit goal of reaching socioeconomically disadvantaged households or areas have achieved greater adoption through various mechanisms, including *a*) a tiered approach offering different stove models and prices for higher- versus lower-income households; *b*) subsidies; *c*) payments in installments; and *d*) access to credit. However, exclusively market-based approaches fail to penetrate beyond a certain level of poverty because disadvantaged groups with limited education tend to perceive other household priorities as being more pressing and therefore tend to generate little or no demand for IS. Consequently, companies usually do not market to “bottom-of-the-pyramid” households unless incentives are provided at the program level.

Women’s decision-making power is often limited because men typically exercise greater budget control. This may be further complicated by women’s distinct gender roles within the household (e.g., first wife, mother-in-law). There appear to be gender-specific preferences with respect to stove attributes, with women valuing health benefits and men favoring fuel efficiency and monetary savings. These should be taken into account in marketing campaigns, where men have been insufficiently targeted to date. Women and women’s networks often play a key role in stove construction and installation, marketing, and sales, although in one Bangladeshi study men were found to be reluctant to support this form of women’s empowerment.

Findings with respect to a programmatic focus on urban versus rural areas are mixed. Commercial businesses, however, tend to target urban areas, where the business is likely to be more feasible and profitable because urban IS users often pay for fuel and are more willing to pay for an improved stove.

## Discussion

*All domains matter*. Drawing on > 50 studies with qualitative, quantitative, and case-study designs, in the present systematic review we identified 31 distinct factors capable of acting as enablers or barriers to the uptake of IS. All seven of the *a priori* defined domains were populated with multiple factors, although some were supported by more evidence than others (Figure 2). This indicates that all domains matter and jointly influence the adoption and sustained use of IS. Integration between factors primarily acting at the household/community level and factors acting primarily at the program/societal level is critical if programs are to reach their intended populations and be successful at scale and over extended periods of time.

Given the many factors identified, an important question is whether some are more important than others and, if so, which. Broadly speaking, the evidence suggests that policies and programs must consider all factors as well as the interrelationships between them. Furthermore, prioritization requires both a suitable method and an evidence base that supports such assessment, and it is not clear that either of these is currently available. Specifically, the heterogeneity inherent in the studies included in the present review makes comparative assessment difficult, with only the quantitative studies using multivariable regression providing any formal analysis of independently associated factors (yet, even then, outcomes vary across studies). Consistency of findings offers some guide to importance, but many factors fulfill this criterion, and a lack of evidence does not mean that a factor is unimportant. For example, few studies report on standards and regulation, but this is mostly a reflection of the historical lack of policy attention in this field. Indeed, much effort is currently being put into developing stove standards with the International Organization for Standardization (ISO) along with establishing regional testing centers (ISO 2012); national regulation governing certification is expected to follow. Consequently, attempts to identify the most important factors are bound to rely mainly on judgment at this stage.

Notwithstanding the constraints on prioritization, it appears that several of the factors across the two household- and community-level domains (notably, socioeconomic aspects, total perceived benefit, and tradition and culture) as well as the fuel and technology characteristics domain (in particular, general design requirements) are likely to act as absolute barriers in all settings; therefore, overlooking them during the design of a policy or program is likely to lead to program failure. For example, an IS that does not meet traditional cooking requirements will not be continuously used, even if a household is initially persuaded to acquire it. Likewise, in accordance with the principles of diffusion of innovation theory (Rogers 1995), perceived overall stove benefits must exceed those of traditional cooking practices for stoves to be adopted and used, even if major contributors can variably be health, fuel, and time savings or social influence. This also illustrates that the importance of many other factors across these three domains tends to vary: For example, the valuation by households of fuel savings and impacts on time differs greatly between settings and social strata.

Insights gathered from a community perspective may provide guidance on where emphasis within the four domains is to be placed at the program/societal level. For example, the identification of a major discrepancy between those technologies that households aspire to have and those they can afford to have will help prioritize stove costs and payment modalities for in-depth consideration and evaluation. As for the technology and household/community level, some factors identified under the financial, tax, and subsidy aspects domain (in particular stove costs and subsidies and program subsidies), the market development domain (notably, demand creation and supply chains), and the programmatic and policy mechanisms domain (importantly, user training and postacquisition support) must be considered in all settings. Some other factors, on the other hand, depend on the technology chosen; installation considerations, for example, are of no concern for portable stoves.

*General and specific insights*. Insights gained from this review have been derived from a mix of smaller-scale projects and programs and a few truly large-scale efforts, including the Chinese and Indian National Improved Stove Programmes (Barnes et al. 2012a, 2012b, 2012c, 2012d, 2012e, 2012f; Sinton et al. 2004). Even though some countries are overrepresented among the included studies, the evidence for 25 of the 31 factors is derived from three continents, suggesting that insights apply globally. It is notable that more or less identical factors emerge independent of scale of delivery, programmatic approach, technology, and household and setting characteristics, although, as described in the section “Equity in adoption and sustained use,” some findings are more relevant to specific population groups such as women, socioeconomically disadvantaged households, or households in urban or rural areas. This reinforces the validity and generalizability of findings, but, as emphasized above, how these factors affect adoption and sustained use of IS is frequently context specific, and detailed mechanisms of action are not necessarily transferable. For example, creating demand among the target population and developing appropriate and reliable supply chains is critical, independent of the policy or programmatic approach. Yet, how incentives for future users are created and whether supply chains are set up exclusively in the private sector or supported by government (e.g., through linkage with ongoing state-supported rural development programs), is highly variable. This context dependence also precludes clear conclusions being drawn in relation to market-based versus state-based approaches. Notably, several factors clearly apply to both philosophies, and future programs can be expected to employ a mix of both market development and government involvement (IEA 2011).

*Intervention effectiveness*. Assessing intervention effectiveness was not among the objectives of this systematic review. Nevertheless, it must be emphasized that the goal of any effort to promote IS should be that households adopt and use the most effective technologies possible. From a health perspective, the impacts on emissions, exposure, and safety are of primary concern. Very few of the included studies provided data on effectiveness or referenced relevant investigations of the technology concerned. However, a growing literature base is showing that the HAP reductions in everyday use and associated health benefits that can be achieved with most currently promoted stove models are limited, at least in terms of reaching levels close to WHO air quality guidelines (Albalak et al. 2001; Hanna et al. 2012; Riojas-Rodríguez et al. 2001), although some chimney stoves have resulted in statistically significant and meaningful health improvements (Chapman et al. 2005; Lan et al. 2002; Shen et al. 2009: Smith et al. 2011). More than half of the studies included in this review promoted at least one stove type with chimneys or smoke hoods but, given model variability and the specific problems reported in relation to chimney installation and maintenance, it cannot be assumed that these stoves resulted in exposure reductions and health benefits similar to those observed in the Guatemalan (Smith et al. 2011) or Chinese studies (Chapman et al. 2005; Lan et al. 2002; Shen et al. 2009). No studies were found on the adoption of recently developed advanced combustion stoves (e.g., forced draft or semi-gasifier stoves). Their low emission rates in the laboratory (relative to more widely used rocket-type stoves) hold future promise (Jetter et al. 2012), although reliable performance in the field remains to be confirmed.

Therefore, a key question is whether findings from the present review on factors influencing uptake of interventions of uncertain effectiveness will be relevant to the adoption of much more effective future interventions. It is likely they are, although some caution is needed. The quality and modernity of stoves and resultant benefits, in particular fuel savings, time savings, cleanliness, and health, are highly valued by users. As these features are strengthened with future technologies, this can be expected to reinforce demand and willingness to pay, as well as longer-term use and maintenance. Another critical and complex issue is the higher price of more advanced stove models. This could exclude low-income households from programs; conversely large-scale production should reduce price, and innovations in financing for both suppliers and potential consumers can effectively extend access.

*Adoption versus sustained use versus exclusive use*. We did not specifically explore factors affecting exclusive or near-exclusive use of IS because this is both a rare phenomenon and rarely studied. Indeed, as previously reported (Ruiz-Mercado et al. 2011) and demonstrated in the present review, adoption does not imply that households abandon their traditional stoves. Factors influencing adoption are likely to differ from those influencing sustained use. Generally speaking, although many stove projects and programs have achieved a reasonable degree of adoption, their sustained use, maintenance, and replacement have been observed less frequently (Table 1). Several factors specifically influence the chances of a program achieving longer-term and sustainable success. For example, insufficient user training, lack of postacquisition support, and nonavailability of stove components limit maintenance and repair of IS. Creating incentives for high-quality maintenance and repair services may be one area where program subsidies can be successfully employed to promote long-term use.

*Methodological strengths and limitations*. This systematic review was broad-based in terms of the range of enablers and barriers considered, short-term versus longer-term uptake assessed, and settings covered, although it focused on household rather than public or commercial settings and excluded refugee camps.

Although carbon-finance studies are likely to provide a rich source of information, none were identified. The present review employed a comprehensive search strategy, combining searches of the peer-reviewed and gray literature with handsearches and expert consultations. This broad approach was critical—all four strategies led to inclusion of studies that would not otherwise have been identified. Screening was undertaken in several languages; we were, however, unable to search Chinese-language databases or screen Chinese-language studies, although we identified a well-known independent evaluation of the NISP. Other aspects of the methodology sought to minimize subjectivity and retain information and referencing to detail (Puzzolo et al. 2013). Consistent findings across settings, research approaches, and study designs represent an important strength. The evidence supporting these factors is, however, drawn from a set of studies that vary greatly in terms of approach, quality, and context. Although the sensitivity analysis suggests that findings are relatively robust with respect to the quality of individual studies, an inherent weakness of our methodological approach is that individual findings become decontextualized. Moreover, studies do not contribute equally toward the insights gained, with “rich” studies reporting findings toward multiple factors across domains, and “poorer” studies—due to the specificity of the research question asked and the nature of the research undertaken—reporting findings toward one or more factors within a single domain.

The validity of the insights gained is fundamentally determined by the quality of the included studies. Established methods were used to appraise individual study quality, although it was sometimes difficult to reliably distinguish between genuinely poor-quality data collection and analysis versus inadequate reporting of methods. A significant proportion of all three study types showed methodological flaws. Despite these flaws, qualitative studies covered context-specific social scenarios offering explanations for why certain factors influence uptake from the perspectives of users. Whereas many quantitative studies did not go beyond simple univariable descriptions of conditions, others used sophisticated modeling approaches to understand the relative impact of various factors. Case studies provided valuable insights through the combination of data and program experience. Whereas some studies, notably quantitative designs carried out at one time of year, may not do justice to the importance of seasonality, insights related to seasonal conditions (e.g., differing household energy needs, value of portability for wet vs. dry seasons) were identified under the relevant factors.

Given the included study designs and their apparent limitations, the majority of individual study findings should be seen as associations rather than as causal effects. It is principally through the combination of studies that conclusions can be drawn about possible causal relationships. Factors that are identified consistently across countries and regions, in distinct types of study, and in an enabling or limiting role are more likely to be causal. For example, postacquisition support is considered essential by users in a large number of qualitative, quantitative, and case studies in Bangladesh, India, and Mexico (Table 2). Where present and of high quality, postacquisition support facilitates regular maintenance and repair; where absent, it reduces stove functionality, with IS falling into disuse and not being maintained, repaired, or replaced as appropriate.

Importantly, by drawing on multiple types of evidence we were able to address the full scope of the systematic review, covering all seven domains as well as equity: Each type of evidence offers explanations that the others are unable to capture. This approach contrasts markedly with a recent systematic review of who adopts IS and clean fuels, which considered only multivariable regression analyses (Lewis and Pattanayak 2012). Based on 11 regression analyses in eight studies and the basic meta-analytical technique of vote counting, Lewis and Pattanayak (2012) found 18 variable groups across the three categories—price, socioeconomic status, and demographics—associated with adoption. Because the authors did not offer any explanation of the likely mechanisms that underlie these associations, it is difficult to draw conclusions with respect to the development of programs and policies. We believe that our approach has led to a more rounded understanding of the factors influencing IS uptake from different perspectives and that it provides a reasonably strong and pragmatic basis for the design and delivery of efforts that are successful at scale and over time.

## Conclusion

In this comprehensive mixed-method systematic review, we identified 31 factors within seven domains that influence the uptake of IS, including equity. Some factors appear to be critical for success, but none can guarantee either adoption or sustained use. Prioritization is problematic given the nature of the available evidence (and potentially the nature of the problem). Therefore, all factors need to be considered, albeit some will be less relevant in certain settings. How then can the findings of this review be implemented in a manner that will help those planning and evaluating programs make the best decisions? We propose two key actions, one relating to the development of a policy tool and one concerning further analysis and future research.

The findings of this review provide the basis for the development of a policy tool to assess all domains and constituent factors, which would be applicable during the planning, implementation, and evaluation stages of policies and programs promoting specific IS in specific settings. The tool could comprise instruments for assessing each relevant factor, and employ a software interface to ensure that unnecessary data collection is avoided.

In terms of future research, we propose two steps. First, the report to the Department for International Development, UK (Puzzolo et al. 2013) includes much more detailed and setting-specific findings, and we would encourage other groups to review this material to identify how implementation could be further enhanced in specific circumstances. Second, research studies are needed to strengthen our understanding of which factors are most important for securing adoption and sustained use, including maintenance and replacement, and will need to draw on a combination of quantitative and qualitative scientific approaches. Qualitative methods can make an important contribution to ensure understanding of the uptake process, in particular by capturing stakeholder perspectives including those of beneficiaries, communities, government, and industry. Prospective evaluations of programs that incorporate the findings of this review (including a focus on complex and controversial topics such as stove subsidies) will be especially useful.

## Supplemental Material

(4.1 MB) PDF
