# Supplementary material for: Enablers and Barriers to Large-Scale Uptake of Improved Solid Fuel Stoves: A Systematic Review
Source: Environ Health Perspect. 2013 Dec 3;122(2):120–30. doi: 10.1289/ehp.1306639 (PMC3914867; doi:10.1289/ehp.1306639)
Supplement: (4.1 MB) PDF [file ehp.1306639.s001.508.pdf]

SUPPLEMENTAL MATERIAL

Enablers and Barriers to Large-Scale Uptake of Improved Solid Fuel Stoves: A Systematic Review

Eva A. Rehfuess, Elisa Puzzolo, Debbi Stanistreet, Daniel Pope, and Nigel G. Bruce

Table of Contents

Supplemental Material, Table S1. Detailed characteristics of qualitative studies, quantitative studies, and case studies (study ID numbers beginning with A, B, and C, respectively) included in the review...2

Supplemental Material, Figure S1. Identification of studies…….……………….……….10

Supplemental Material, Figure S2. Graphical sensitivity analysis: Factors influencing uptake of improved solid fuel stoves based on moderate- and high-quality studies…………………11

Supplemental Material, References…………………………………………………...…12

Supplemental Material, Table S1. Detailed characteristics of qualitative studies, quantitative studies, and case studies (study ID numbers beginning with A, B, and C, respectively) included in the review.

ID	Source	Country/Setting	Study design and sampling	Data collection	Data analysis	Quality appraisal[a]	Improved stove technology: stove type, number of potholes, stove ventilation	Stove production	Adoption (A) vs. sustained use (S)
A1	Anderson 2007	India. Maharashtra state (rural)	Ethnographic case study: 3 FGDs (n=7-8 each), 3 SSIs, 2 KIIs, PO	Interviews and FGDs with women users and non-users	Editing analysis	Strong	Bhagyalaxmi stoves (cement), 2 potholes, unvented	Centrally produced (assumed)	S
A2	Chowdhury et al. 2011	Bangladesh, Habigonj region (rural)	70 SSIs, 1 FGD (n=unknown), PO	Face-to-face survey and FGD with women users and non-users	Method not stated; descriptive narrative and tables	Weak	Mud stoves, 2 potholes, vented	Locally produced (assumed)	A/S
A3	Christoff 2010	Mexico, State of Mexico (rural)	4 FGDs (n=9-14 each)	FGDs with women users	Thematic analysis	Strong	Patsari stoves, multiple potholes, vented; Onil stoves, 1 pothole, vented	Not specified	A
A4	Gordon et al. 2007	Mongolia, city of Ulaanbaatar (urban)	3 FGDs (n=8 each), 6 SSIs	Mixed-gender FGDs with users and non-users	Editing analysis	Strong	Coal stoves, vented	Not specified	A
A5	Jagoe et al. 2006a, Qualitative findings	India, Bundelkhand region (rural)	Mixed method approach: 11 FGDs at baseline and 8 FGDs at 12- month follow-up; 3 KIIs	Separate FGDs with men and women users	Framework analysis	Moderate	Anandi stoves, 1 pothole, vented; Sukhad stoves, 2 potholes, vented	Not specified	A
A6	Jagoe et al. 2007a, Qualitative findings	India, Maharashtra state (rural)	Mixed method approach: FGDs at baseline and after 6 and 12 months follow-up (n=unknown), 2 KIIs	FGDs with women users and non-users	Framework analysis	Moderate	Bhagyalaxmi stoves, 2 potholes, unvented; Laxmi stoves, 2 potholes, vented	Not specified	A
A7	Pandey 1989	Nepal, Dhading district (rural)	Mixed method approach: 25 SSIs, PO	Interviews with women users and non-users	Method not stated; descriptive narrative	Moderate	Bikase stoves, 2 potholes, unvented	Not specified	A/S

ID	Source	Country/Setting	Study design and sampling	Data collection	Data analysis	Quality appraisal[a]	Improved stove technology: stove type, number of potholes, stove ventilation	Stove production	Adoption (A) vs. sustained use (S)
A8	Person et al. 2012	Kenya, Nyanza province (rural)	40 SSIs	Interviews with purchasers and stove promoters	Thematic analysis	Strong	Upesi Jiko charcoal stoves, 1 pothole, unvented	Locally produced	A
A9	Sesan 2012	Kenya, Western Kenya (urban[b])	15 SSIs, 9 KIIs, PO	Interviews with women users and stakeholders	Method not stated; descriptive narrative	Moderate	Mainly Upesi Jiko charcoal stoves, 1 pothole, vented or unvented	Not specified	A
A10	Simon 2007	India, Maharashtra state (rural)	55 SSIs, 11 KIIs, PO	Interviews with women users, stove builders and stakeholders	Method not stated; descriptive narrative	Strong	Bhagylaxmi and Laxmi stoves (cement), 1 or 2 potholes, vented or unvented; other stove models	Locally produced	A/S
A11	Sovacool and Drupady 2011	Bangladesh, countrywide (rural/urban)	Case study based on 48 SSIs/KIIs	Interviews with users and stakeholders	Narrative analysis	Moderate	Clay stoves, 1, 2 or 3 potholes, vented	Locally produced (assumed)	A/S
A12	Troncoso et al. 2007	Mexico, Michoacán state (rural)	67 SSIs, 18 KIIs	Interviews with women users and non-users	Method not stated; descriptive narrative and tables	Moderate	Patsari stoves, multiple potholes, vented	Locally produced (assumed)	A
A13	Troncoso et al. 2011	Mexico, Michoacán state (rural)	24 KIIs	Interviews with stakeholders, including stove builders	Method not stated; descriptive narrative	Moderate	Patsari stoves (three different models), multiple potholes, vented	Locally produced (assumed)	A
A14	Velasco 2008	Mexico, Michoacán state (rural)	24 SSIs	Interviews with women users	Method not stated; descriptive narrative	Moderate	Patsari stoves, multiple potholes, vented	Locally produced (assumed)	A
B1	Agurto-Adrianzen 2009	Peru, Chalaco district (rural)	Cross-sectional survey (n=816); stove monitoring survey (n=82% of beneficiaries)	Interviews with heads of household (users/non-users)	Multivariable approach adjusting for confounders	Strong	Mud brick and metal frame/plate stoves, multiple potholes, vented	Locally produced	A
B2	Bensch and Peters 2011	Senegal, cities of Dakar and Kaolack (urban)	Cross-sectional survey (n=624)	Interviews with user/non-users	Analytical approach without adjustment	Moderate	Portable Jambar charcoal stoves (metal with clay inlay), unvented	Centrally produced (assumed)	A

ID	Source	Country/Setting	Study design and sampling	Data collection	Data analysis	Quality appraisal[a]	Improved stove technology: stove type, number of potholes, stove ventilation	Stove production	Adoption (A) vs. sustained use (S)
B3	Damte and Koch 2011	Ethiopia, Amahra, Oromiya and Tigray regions (urban)	Cross-sectional survey (n=1577)	Interviews with user/non users	Multivariable approach adjusting for confounders	Strong	Mirt Injera stoves (cement and pumice), 1 pothole, unvented; portable Lakech charcoal stoves (clay, sand, cement and metal for cladding)	Not specified	S
B4	El Tayeb Muneer and Mohamed 2003	Sudan, Khartoum state (rural/urban)	Cross-sectional survey (n=300)	Interviews with wife and husband in household	Multivariable approach adjusting for confounders	Strong	Firewood/charcoal stoves	Not specified	A
B5	George and Yadla 1995	India, Gujarat state (rural)	Cross-sectional survey (n=390)	Interviews with main cooks	Descriptive comparison and analytical approach without adjustment	Weak	Mamta stoves (mud, brick), 2 potholes, vented	Not specified	A
B6	Inayatullah 2011	Pakistan, Swat district (rural)	Cross-sectional survey (n=100)	Interviews with male respondents	Multivariable logistic regression	Moderate	Biomass metal stoves, 1 pothole, unvented	Locally produced	A
B7	Jagoe et al. 2006b, Quantitative findings	India, Bundelkhand region (rural)	Before-and after-study (12 months) without control group (n=150)	Structured questionnaires at baseline, follow-up at 6 and 12 months	Descriptive comparison	Weak	Anandi stoves, 1 pothole, vented; Sukhad stoves, 2 potholes, unvented	Not specified	A
B8	Jagoe et al. 2007b, Quantitative findings	India, Maharashtra state (rural)	Before-and after-study (12 months) with control group (n=156 nterventions, n=98 controls)	Structured questionnaires at baseline, follow-up at 6 and 12 months	Multivariable approach adjusting for confounders	Moderate	Bhagyalaxmi stoves, 2 potholes, unvented; Laxmi stoves, 2 potholes, vented	Not specified	A
B9	Levine and Cotterman 2012	Uganda, city of Kampala (urban)	Randomized trial of multiple sale offers (n=1690)	Interviews during marketing visits	Multivariable approach adjusting for confounders	Moderate	Ugastove charcoal stoves (metal), 1 pothole, unvented	Centrally produced (assumed)	A
B10	Miller and Mobarak 2011	Bangladesh, Jamalpur, and Haita districts (rural)	Randomized controlled trial (n=3079)	Interviews during marketing visits	Multivariable approach adjusting for confounders	Strong	Mud stoves, 1 pothole, unvented; clay stoves, 2 potholes, vented	Locally produced	A

ID	Source	Country/Setting	Study design and sampling	Data collection	Data analysis	Quality appraisal[a]	Improved stove technology: stove type, number of potholes, stove ventilation	Stove production	Adoption (A) vs. sustained use (S)
B11	Mwangi 1992	Kenya, Nyeri district (rural)	Cross-sectional survey (n=306)	Interviews with heads of household	Multivariable approach adjusting for confounders	Moderate	Kenya Ceramic Jiko charcoal stoves, 1 pohole, unvented; portable Kuni Mbili stoves (ceramic), 1 pothole, unvented	Not specified	A
B12	Pandey and Yadama 1992	Nepal, Dhading district (rural)	Cross-sectional survey (n=100)	Interviews with women	Analytical approach without adjustment	Weak	Bikase stoves, 2 potholes, unvented	Not specified	A
B13	Pine et al. 2011	Mexico, Michoacan state (rural)	Longitudinal study with baseline survey and monthly follow-up surveys over 10 months (n=233)	Interviews with users only	Univariate multinomial logistic regression	Moderate	Patsari stoves, 3 potholes, vented	Locally produced (assumed)	A
B14	Pushpa 2011	India, Southern region (rural)	Cross-sectional survey (n=492)	Interviews with users/non users	Analytical approach without adjustment	Weak	Several stove models, vented	Not specified	A
B15	Silk et al. 2012	Kenya, Nyanza province (rural)	Cross-sectional survey (n=1250) and follow-up (n=293)	Interviews with women; only purchasers interviewed at follow-up	Analytical approach without adjustment	Moderate	Upesi Jiko biomass and charcoal stoves (ceramic), 1 pothole, unvented	Locally produced	A
B16	Wallmo and Jacobson 1998	Uganda, Western region (rural)	Cross-sectional survey (n=165)	Interviews with users/non-users	Descriptive comparison and analytical approach without adjustment	Weak	Lorena stoves (mud), 3 potholes, vented	Locally produced	A
C1	Amarasekera 1989	Sri Lanka, countrywide (rural/urban)	Surveys (n=not stated)	Not described	Descriptive narrative	Weak	Mud stoves, 1 or 2 potholes, unvented	Locally produced (assumed)	A

ID	Source	Country/Setting	Study design and sampling	Data collection	Data analysis	Quality appraisal[a]	Improved stove technology: stove type, number of potholes, stove ventilation	Stove production	Adoption (A) vs. sustained use (S)
C2	Barnes et al. 2012a	India, Western Maharashtra state (rural/urban)	Mixed method approach: Household survey (n=73) and FGDs, SSIs and KIIs (n=unknown)	Interviews and discussions with users and non-users, stove builders and other stakeholders	Descriptive narrative and statistics	Strong	Laxmi stoves (mud), 2 potholes, vented; Grihalaxmi, Parvati and Bhagyalaxmi stoves (mud), 1 or 2 potholes, unvented; portable Priagni stoves (metal), 1 pothole, unvented	Locally produced	S
C3	Barnes et al. 2012b	India, Haryana state (rural/urban)	Mixed method approach: Household survey (n=94) and FGDs, SSIs, KIIs (n=unknown)	Interviews and discussions with users and non-users, stove builders and other stakeholders	Descriptive narrative and statistics	Strong	Mohin, Mohini Hara and Jaitan stoves (mud), 1 or 2 potholes, vented; Akash and Sohini Hara stoves (cement), 1 or 2 potholes, vented	Locally produced	S
C4	Barnes et al. 2012c	India, Karnataka state (rural/urban)	Mixed method approach: Household survey (n=190), FGDs (n=217 women), SSIs and KIIs (n=unknown)	Interviews and discussions with users and non-users, stove builders and other stakeholders	Descriptive narrative and statistics	Strong	Sarale Ole (mud), Sukhad, Mamatha and Abhinava stoves, 1 or 2 potholes, vented and unvented; portable Priagni stoves (ceramic), 1 pothole, unvented; portable Swosthee and Chara Ole (metal) stoves, 1 pothole, unvented	Locally produced	S
C5	Barnes et al. 2012d	India, Gujarat state (rural/urban)	Mixed method approach: Household survey (n=79) and FGDs, SSIs, KIIs (n=unknown)	As above	Descriptive narrative and statistics	Strong	Mamta, Supriya, Priya, Kiran, Sneha and Kamdhenu stoves, 1 or 2 potholes, vented; Grihalaxmi stoves, 1 pothole, unvented	Locally produced	S
C6	Barnes et al. 2012e	India, Andhra Pradesh state (rural/urban)	Mixed method approach: Household survey (n=134) and FGDs, SSIs, KIIs (n=unknown)	As above	Descriptive narrative and statistics	Strong	Sukhad, Gayathri stoves (brick/mud or cement), 1 or 2 potholes, vented; Gramalakshmi stoves (mud), 2 potholes, unvented	Locally produced	S

ID	Source	Country/Setting	Study design and sampling	Data collection	Data analysis	Quality appraisal[a]	Improved stove technology: stove type, number of potholes, stove ventilation	Stove production	Adoption (A) vs. sustained use (S)
C7	Barnes et al. 2012f	India, West Bengal state (rural/urban)	Mixed method approach: Household survey (n=100) and FGDs, SSIs, KIIs (n=unknown)	As above	Descriptive narrative and statistics	Strong	Sohini, Sugam, Paribarbandhu stoves (mud or cement), 1 or 2 potholes, vented; Kalyani biomass and coal stoves (mud), 1 pothole; Kalyani Vishwavidyalaya stoves (mud), 1 pothole, unvented	Locally produced	S
C8	GERES 2009	Cambodia, Kampong Chhanang province (urban)	Mixed method approach: Cross-sectional survey (n=1600) and 51 SSIs	Interviews with users and stove builders	Descriptive narrative and statistics	Strong	New Lao charcoal stoves (clay), 1 pothole, unvented	Locally produced	A/S
C9	Kürschner et al. 2009	Bangladesh, countrywide (rural/urban)	Mixed method approach: Surveys, FGDs and interviews (450 participants in total)	Interviews with users, non-users and stove builders	Descriptive narrative	Moderate	Mud/clay stoves, 1 or 2 potholes, vented	Locally produced (assumed)	A/S
C10	Masera et al. 2005	Mexico, Michoacan state (rural)	Cross-sectional survey (n=42)	Interviews with users	Descriptive narrative	Moderate	Patsari stoves, multiple potholes, vented	Locally produced	A
C11	Mounkalia 1989	Niger, city of Niamey (urban)	Surveys (n=1000) and KIIs	Questionnaire administered to users and non-users	Descriptive narrative and statistics	Weak	Mai Sauki metal stoves, 1 pothole, unvented	Centrally produced (assumed)	A
C12	Namuye 1989	Kenya, cities of Nairobi and Kisumu (urban)	Survey (n=>500 households)	Interviews with users, stove producers and stove promoters	Descriptive narrative	Weak	Kenya Ceramic Jiko charcoal stoves, 1 pothole, unvented	Centrally produced (assumed)	A
C13	Osei 2010	Ghana, countrywide (rural/urban)	Business model case study (3 KIIs)	Not described	Descriptive narrative	Weak	Toyola charcoal stoves (ceramic, metal), 1 pothole, unvented	Centrally produced	A
C14	Sawadogo 1989	Burkina Faso, city of Ouagadougou (urban)	Mixed method approach: Survey, interviews (2 households per district), PO	Face-to-face interviews. Three days spent within each households	Descriptive narrative and statistics	Weak	Ouaga stoves (ceramic, metal) and Mixte wood or charcoal stoves, 1 pothole, unvented	Centrally produced (assumed)	A

ID	Source	Country/Setting	Study design and sampling	Data collection	Data analysis	Quality appraisal[a]	Improved stove technology: stove type, number of potholes, stove ventilation	Stove production	Adoption (A) vs. sustained use (S)
C15	Shastri et al. 2002	India, Karnataka state (rural)	Cross-sectional surveys (n=155 in 1994 and n=132 in 2001)	Interviews with housewives	Descriptive narrative and statistics	Strong	Astra stoves (mud), 2 or 3 potholes, vented	Not specified	S
C16	Shrimali et al. 2011	India, countrywide (rural/urban)	12 KIIs	Interviews with company representatives	Descriptive narrative and statistics	Strong	Several stove models	Not specified	S
C17	Simon 2010	India, Western Maharashtra state (rural)	55 SSIs, surveys, 11 KII, PO	Interviews with women users, stove builders and NGO employees	Descriptive narrative	Moderate	Laxmi and Bhagylaxmi stoves (cement), 1 or 2 potholes, vented or unvented; other stove models	Locally produced	A/S
C18	Sinton et al. 2004	China, countrywide (rural/urban)	Mixed method approach: Household survey (n=3476) and facility survey with stakeholders (n=108)	Open-ended interviews with structured questionnaire	Descriptive narrative and statistics	Strong	Biomass and coal stoves, multiple potholes, vented	Locally produced (assumed)	S
C19	Sudjarwo et al. 1989	Indonesia, Sleman and Bantul areas (rural)	Surveys of users and non-users (n=1000) and PO	Interviews with households, stove producers and stove traders	Descriptive narrative and statistics	Weak	SAE pottery stoves (clay), 2 potholes, unvented	Not specified	A/S
C20	USAID/ Winrock 2008	Peru, Lambayeque region (rural)	Mixed method approach: Survey (n=169) and FGDs (n=unknown)	Not described	Descriptive narrative and statistics	Moderate	Inkawasina rocket stoves, 2 potholes, vented	Locally produced	S
C21	USAID/ Winrock 2009	Bangladesh, cities of Saidpur and Parbatipur (urban)	Survey (n=625)	Interviews with main cooks	Descriptive narrative	Moderate	Portable and fixed BCSIR stoves, 1 or 2 potholes, vented; Grihalaxmi stoves, 1 pothole, unvented	Locally produced (assumed)	A
C22	World Bank 2004a	Guatemala, Baja Verapaz department (rural)	24 SSIs, 2 FGDs (n=8-12 each)	Interviews and discussions with users	Descriptive narrative and statistics	Moderate	Tezulutlan plancha stoves (brick, clay), 3 potholes, vented	Locally produced	A
C23	World Bank 2004b	Guatemala, Jalapa department (rural)	31 SSIs and 2 FGD (n=12-14)	Interviews and discussions with users	Descriptive narrative and statistics	Moderate	Plancha stoves (brick), 1, 2, 3 or 4 potholes, vented	Locally produced	A

ID	Source	Country/Setting	Study design and sampling	Data collection	Data analysis	Quality appraisal[a]	Improved stove technology: stove type, number of potholes, stove ventilation	Stove production	Adoption (A) vs. sustained use (S)
C24	World Bank. 2004c	Guatemala, Western Guatemala (rural)	32 SSIs and 2 FGDs (n=6-8 each)	Interviews and discussions with users	Descriptive narrative and statistics	Moderate	Plancha stoves (brick), 3 potholes, vented	Locally produced	A
C25	World Bank 2010a	Bangladesh, 28 districts and cities of Dhaka and Rajshashi (rural/urban)	Literature review supported by surveys (n=142) and 41 FGDs and KIIs[c]	Survey with users, interviews with technicians and stakeholders	Descriptive narrative	Moderate	Portable or semi-submerged mud stoves, 1 pothole, unvented; fixed mud stoves, 1 or 2 potholes, vented	Locally produced (assumed)	S
C26	World Bank 2010b	Bangladesh, countrywide (rural/urban)	Literature review supported by surveys (n=142) and 41 FGDs and KIIs[c]	Survey with users, interviews with technicians and stakeholders	Descriptive narrative	Moderate	Mud or mud/brick stoves, 1 or 3 potholes, vented	Locally produced (assumed)	S
C27	World Bank 2010c	Bangladesh, municipalities of Saidpur and Parbatipur (urban)	Literature review supported by surveys (n=142) and 41 FGDs and KIIs[c]	Survey with users, interviews with technicians and stakeholders	Descriptive narrative	Moderate	Portable and fixed BCSIR stoves, 1 or 2 potholes, vented; Grihalaxmi stoves, 1 pothole, unvented	Locally produced (assumed)	A

FDG = Focus group discussion; SSI =Semi-structured interview, KII = Key informant interview, PO = Participant observation.

[a]Quality appraisal of studies was conducted using three separate quality assessment tools resulting in an overall score of strong, moderate or weak. It is, however, important to note that quality appraisal across study designs is not directly comparable. [b]This study was conducted in a peri-urban setting. [c]These figures are cumulative for World Bank 2010a, World Bank 2010b and World Bank 2010c; a breakdown for each of the three case studies is not available.

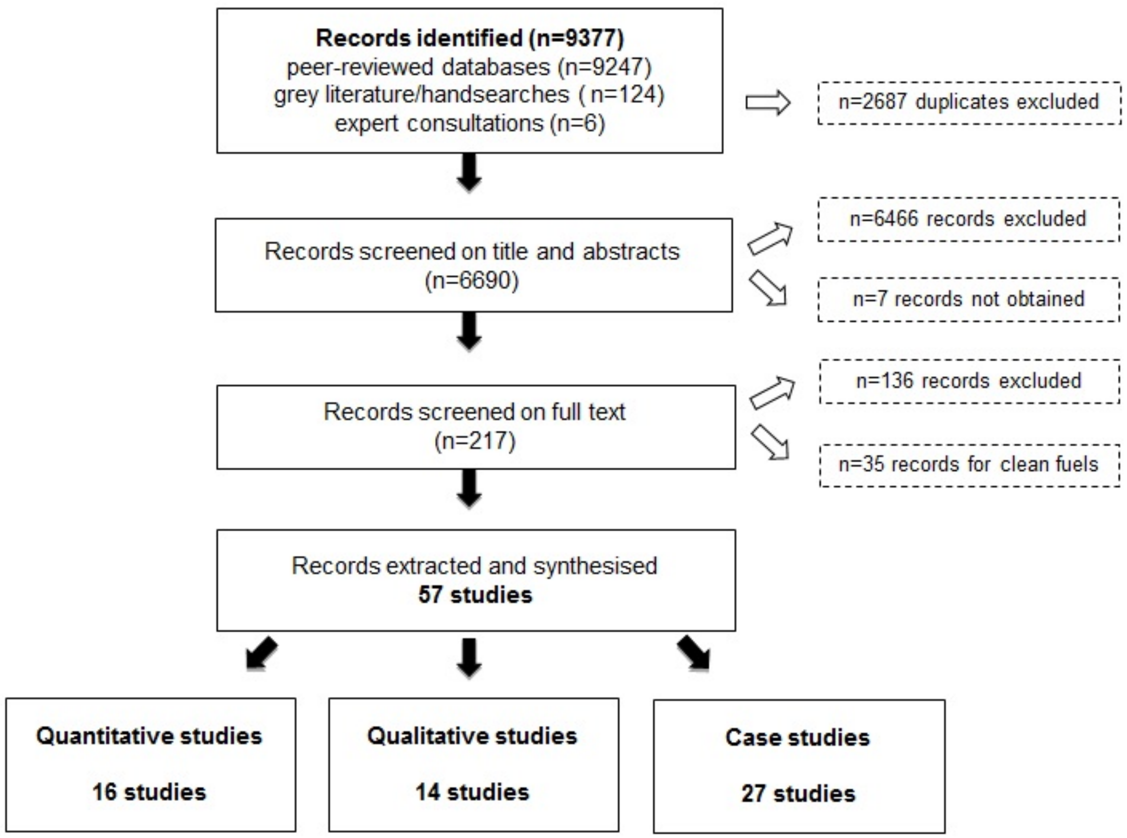

Supplemental Material, Figure S1. Identification of studies.

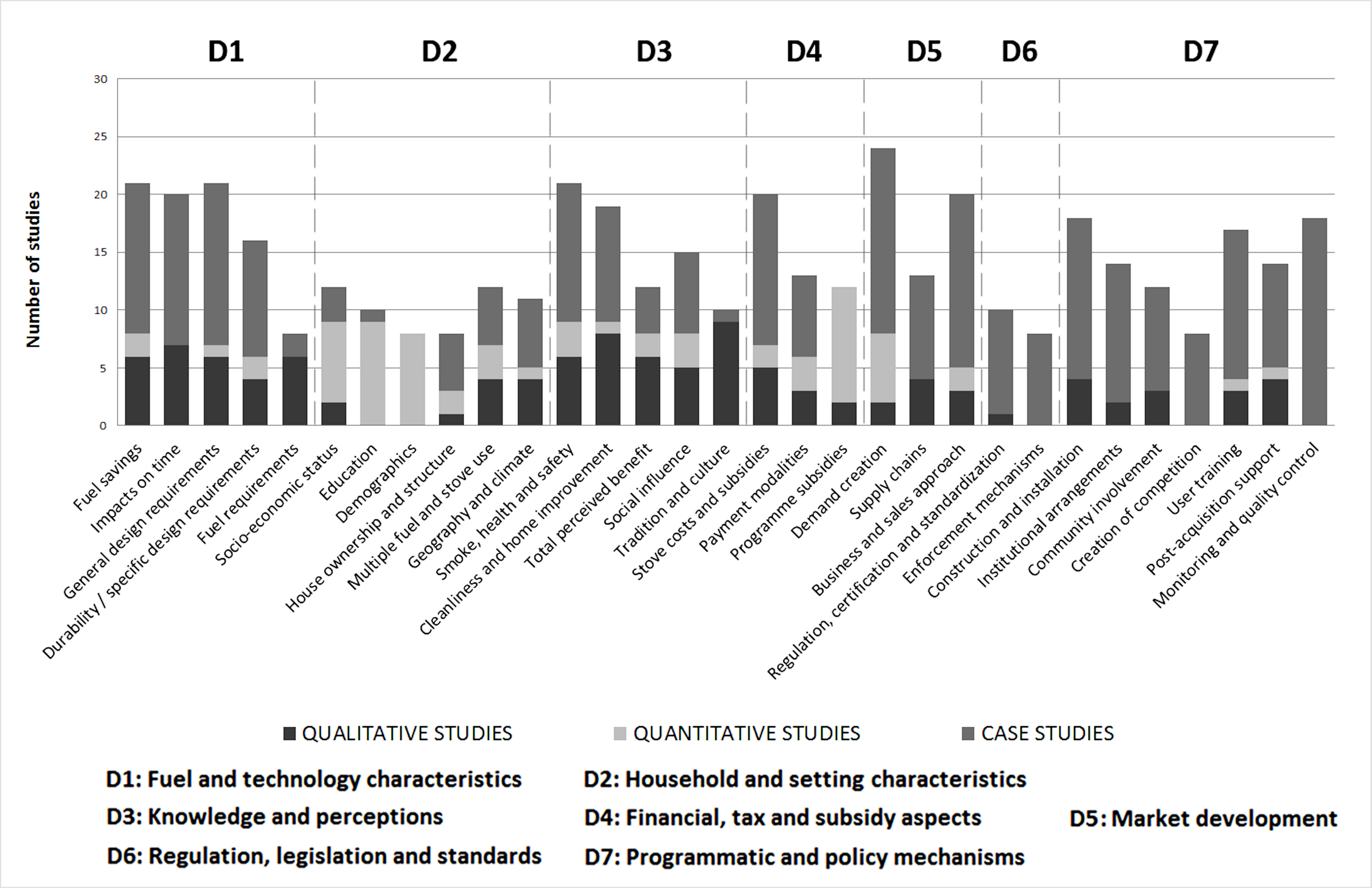

Supplemental Material, Figure S2. Graphical sensitivity analysis: Factors influencing uptake of improved solid fuel stoves based on moderate- and high-quality studies.

References

Agurto-Adrianzen M. 2009. The role of social capital in the adoption of firewood efficient stoves in Northern Peruvian Andes. MPRA Paper No. 15918. Munich: Munich Personal RePEc Archive. Available: http://mpra.ub.uni-muenchen.de/15918/1/MPRA_paper_15918.pdf [accessed 1 August 2013].

Amarasekera RM. 1989. Sri-Lanka: Status of Improved Woodstove Dissemination in Sri Lanka. In: Cáceres R (ed.) Stoves for People. Proceedings of the 2nd International Workshop on Stoves Dissemination, 4-10 October 1987, Antigua, Guatemala. Exeter: IT Publications, pages 118-121. Available: http://search.library.wisc.edu/catalog/ocm22748697 [accessed 20 November 2013].

Anderson ZC. 2007. Reducing indoor air pollution on developing countries: a case study investigating the utilization of improved stoves in rural India. Available: http://www.hedon.info/docs/Acasestudyinvestigatingtheutilizationofimprovedsto%5B1%5D..pdf [accessed 20 November 2013].

Barnes DF, Kumar P, Openshaw K. 2012a. Maharashtra - commercial approach. Chapter 3 in: Cleaner hearths, better homes: new stoves for India and the developing world. New York: Oxford University Press, pages 24-35.

Barnes DF, Kumar P, Openshaw, K. 2012b. Haryana - women's involvement approach Chapter 4: Cleaner hearths, better homes: new stoves for India and the developing world.: New York: Oxford University Press, pages 36-49.

Barnes DF, Kumar P, Openshaw, K. 2012c. Karnataka - Technical innovation and institutions. Chapter 5 in: Cleaner hearths, better homes: new stoves for India and the developing world. New York: Oxford University Press, pages 50-63.

Barnes DF, Kumar P, Openshaw K. 2012d. Gujarat - rural development approach. Chapter 6 in: Cleaner hearths, better homes: new stoves for India and the developing world. New York: Oxford University Press, pages 64-77.

Barnes DF, Kumar P, Openshaw K. 2012e. Andhra Pradesh - interagency coordination. Chapter 7 in:. Cleaner hearths, better homes: new stoves for India and the developing world.New York: Oxford University Press, pages 78-94.

Barnes DF, Kumar P, Openshaw K. 2012f. West Bengal – Nongovernmental organizations. Chapter 8 in: Cleaner hearths, better homes: new stoves for India and the developing world. New York: Oxford University Press, pages 95-113.

Bensch G, Peters J. 2011. Combating deforestation? Impacts of improved stove dissemination on charcoal consumption in urban Senegal. RUHR Economics Papers 306. Essen: Rheinisch-Westfälisches Institut für Wirtschaftsforschung. Available: http://www.rwi-essen.de/media/content/pages/publikationen/ruhr-economic-papers/REP_11_306.pdf [accessed 20 November 2013].

Chowdhury MSH, Koike M, Akther S, Miah MD. 2011. Biomass fuel use, burning technique and reasons for the denial of improved cooking stoves by Forest User Groups of Rema-Kalenga Wildlife Sanctuary, Bangladesh. Int J Sustain Dev World Ecol 18(1):88-97.

Christoff J. 2010. Benefits and barriers: exploring complete and sustained ecological stove usage in rural Mexico. MPH thesis. New Haven, CT: Yale University. Available: http://gradworks.umi.com/14/80/1480293.html [accessed 20 November 2013].

Damte A, Koch SF. 2011. Clean fuel saving technology adoption in urban Ethiopia. Department of Economics Working Paper Series. Pretoria: University of Pretoria.

El Tayeb Muneer S, Mukhtar Mohamed EW. 2003. Adoption of biomass improved cookstoves in a patriarchal society: An example from Sudan. Sci Total Environ 307(1-3):259-66.

George R, Yadla V. 1995. Factors affecting perception of beneficiaries of National Programme on Improved Cookstoves regarding cost-benefit of adoption of Mamta stove. In: Proceedings of the Annual Conference of the American Solar Energy Society, 15-20 July 1995, Minneapolis, Minnesota: Boulder, CO: American Solar Energy Society, 361-366.

GERES. 2009. Dissemination of domestic efficient cookstoves in Cambodia. Aubagne: Groupe Energies Renouvelables, Environnement et Solidarités. Available: http://www.geres.eu/images/stories/publis/publi-nls-en.pdf [accessed 2 April 2013].

Gordon JK, Emmel ND, Manaseki S, Chambers J. 2007. Perceptions of the health effects of stoves in Mongolia. J Health Organ Manag 21(6):580-587.

Inayatullah J. 2011. What makes people adopt improved cookstoves? Empirical evidence from rural Northwest Pakistan. The Governance of Clean Development

Working Paper Series 012. Norwich: University of East Anglia. Available: http://www.uea.ac.uk/international-development/research/gcd/Inayat+2011 [accessed 2 April 2013]

Jagoe K, Bromley H, Chengappa C, Bruce NG. 2006. Evaluation of the health and socio-economic impacts of Development Alternatives pilot project Energy Services for Village Households and Rural Enterprises in Bundelkhand, India. Final report. Available upon request from Nigel Bruce, ngb@liverpool.ac.uk

Jagoe K, Bromley H, Dutta K, Bruce NG. 2007. Standard monitoring packages for household energy and health field projects. Final report. Available upon request from Nigel Bruce, ngb@liverpool.ac.uk

Kürschner E, Diehl E, Hermann-Friede J, Hornikel C, Rosenbusch J et al. 2009. Impact of basic rural energy services in Bangladesh. SLE Publication Series No. S238. Berlin: Humboldt Universität zu Berlin. Available: http://edoc.hu-berlin.de/series/sle/238/PDF/238.pdf [accessed 2 April 2013].

Levine DI, Cotterman C. 2012. What impedes efficient product adoption? Evidence from randomized variation in sales offers for improved cookstoves in Uganda. Working Paper Series, Institute for Research on Labor and Employment. San Francisco, CA: University of California at Berkeley. Available: http://www.cleancookstoves.org/resources_files/what-impedes-efficient.pdf [accessed 2 April 2013].

Masera OR, Díaz R, Berrueta V. 2005. From cookstoves to cooking systems: the integrated program on sustainable household energy use in Mexico. Energy for Sustain Dev 9(1):25-36.

Miller G, Mobarak MA. 2011. Gender differences in preferences, intra-household externalities and low demand for a new technology: experimental evidence on improved cookstoves. NBER Working Paper Series No. 18964. Cambridge, MA: National Bureau of Economic Research. Available: http://www.nber.org/papers/w18964 [accessed 2 April 2013].

Mounkalia A. 1989. Niger: The promotion and dissemination of improved stoves. In: Cáceres R (ed.) Stoves for People. Proceedings of the 2nd International Workshop on Stoves Dissemination, pages: 4-10 October 1987, Antigua, Guatemala. Exeter: IT Publications,46-50. Available: http://search.library.wisc.edu/catalog/ocm22748697 [accessed 20 November 2013].

Mwangi AM. 1992. Analysis of wood energy production and consumption strategies among small-scale farmers in central Kenya. Ph.D. thesis. East Lansing, MI: Michigan State University.

Namuye SA. 1989. Survey on dissemination and impact of Kenya Ceramic Jiko in Kenya. In: Cáceres R (ed.) Stoves for People. Proceedings of the 2nd International Workshop on Stoves Dissemination, 4-10 October 1987, Antigua, Guatemala. Exeter: IT Publications, pages 40-44. Available: http://search.library.wisc.edu/catalog/ocm22748697 [accessed 20 November 2013].

Osei, Robert D. 2010. Toyola Charcoal Stove: Improving the Environment and Health of the Poor in Ghana. GIM Case Study No. B095. New York: United Nations Development Programme. Available: http://www.growinginclusivemarkets.org/media/cases/Ghana_Toyola_2010.pdf [accessed 20 November 2013].

Pandey S. 1989. Some factors determining level of use of improved stoves by Brahmin and Chhetri women in Central Nepal. Ph.D. thesis. Cleveland, Ohio: Case Western Reserve University.

Pandey S, Yadama GN. 1992. Community development programs in Nepal: a test of diffusion of innovation theory. Soc Serv Rev 66(4):582-582.

Person B, Loo JD, Owuor M, Ogange L, Jefferds ME, Cohen AL. 2012. "It is strong for my family's health and cooks food in a way that my heart loves": Qualitative findings and implications for scaling up an improved cookstove project in rural Kenya. Int J Environ Res Public Health 9:1566-1580.

Pine K, Edwards R, Masera OR, Schilmann A, Marrón-Mares A, Riojas-Rodríguez H. 2011. Adoption and use of improved biomass stoves in rural Mexico. Energy Sustain Dev 15(2):176-83.

Pushpa, K. 2011. Factors associated with the adoption of improved cook stoves in Southern parts of India. International Symposium on Humanities, Science and Engineering Research, 6-7 June 2011, Kuala Lumpur, Malaysia. Available: http://ieeexplore.ieee.org/xpls/abs_all.jsp?arnumber=6008490 [accessed 1 August 2013].

Sawadogo A. 1989. Fuelwood consumption and improved stoves diffusion in Ouagadougou City. In Cáceres R (ed.): Stoves for People. Proceedings of the 2nd International Workshop on Stoves Dissemination, 4-10 October 1987, Antigua, Guatemala. Exeter: IT Publications, pages 3-9. Available: http://search.library.wisc.edu/catalog/ocm22748697 [accessed 20 November 2013].

Sesan TA. 2012. Navigating the limitations of energy poverty: lessons from the promotion of improved cooking technologies in Kenya. Energy Policy 47:202-210.

Shastri CM, Sangeetha G, Ravindranath NH. 2002. Dissemination of efficient ASTRA stove: case study of a successful entrepreneur in Sirsi, India. Energy Sustain Dev 6(2):63-67.

Shrimali G, Slaski X, Thurber MC, Zerriffi H. 2011. Improved stoves in India: A study of sustainable business models. Energy Policy 39(12):7543–7556.

Silk B, Sadumah I, Patel M, Were V, Person B et al. 2012. A strategy to increase adoption of locally-produced, ceramic cookstoves in rural Kenyan households. BMC Public Health 12:359.

Simon G. 2007. Brokering development: Geographies of mediation and energy sector reforms in Maharashtra, India. Ph.D. thesis. Seattle, WA: University of Washington.

Simon G. 2010. Mobilizing cookstoves for development: a dual adoption framework analysis of collaborative technology innovations in Western India. Environ Planning 42(8):2011-2030.

Sinton JE, Smith KR, Peabody JW, Yaping L, Xiliang Z, Edwards R, Quan G. 2004. An assessment of programs to promote improved household stoves in China. Energy Sustain Dev 8(3):33-52.

Sovacool BK, Drupady IM. 2011. Summoning earth and fire: The energy development implications of Grameen Shakti (GS) in Bangladesh. Energy 36:4445-59.

Sudjarwo, A, Herm UY, Suryaningati D, Sumarni, Sunarno. 1989. Indonesia: Pottery stoves, their production, dissemination and adoption. In Cáceres R (ed.): Stoves for People. Proceedings of the 2nd International Workshop on Stoves Dissemination, 4-10 October 1987, Antigua, Guatemala. Exeter: IT Publications, pages 32-38. Available: http://search.library.wisc.edu/catalog/ocm22748697 [accessed 20 November 2013].

Troncoso K, Castillo A, Masera OR, Merino L. 2007. Social perceptions about a technological innovation for fuelwood cooking: case study in rural Mexico. Energy Policy 35(5):2799-2810.

Troncoso K, Castillo A, Merino L, Lazos, Masera OR. 2011. Understanding an improved cookstove program in rural Mexico: an analysis from the implementers' perspective. Energy Policy 39(12):7600-08.

USAID/Winrock. 2008. Peru healthy kitchen healthy stove pilot project. Washington DC: United States Agency for International Development. Available: http://pdf.usaid.gov/pdf_docs/PDACN009.pdf [accessed 2 April 2013].

USAID/Winrock. 2009. Commercialization of improved cookstoves for reduced indoor air pollution in urban slums of northwest Bangladesh. Washington DC: United States Agency for International Development. Available: http://pdf.usaid.gov/pdf_docs/pnado851.pdf [accessed 2 April 2013].

Velasco, I. 2008. More sustainable cooking technologies: a case study in rural kitchens in Michoacan. M.Sc. thesis. Lund: Lund University. Available: http://www.lumes.lu.se/database/alumni/06.08/thesis/Ignacio_Velasco.pdf [accessed 20 November 2013].

Wallmo K, Jacobson SK. 1998. A social and environmental evaluation of fuel-efficient cook-stoves and conservation in Uganda. Environ Conserv 25(2):99-108.

World Bank. 2004a. Case Study 1: Tezulutlan project. In: Evaluation of improved stove programs in Guatemala: final report of project case studies. ESMAP Technical Paper No. 60. Washington DC: World Bank, pages 21-44. Available: http://www.esmap.org/sites/esmap.org/files/06004GuatemalaFinalEnglishforWeb.pdf [accessed 20 November 2013].

World Bank. 2004b. Case Study 2: Social Investment Fund Project. In: Evaluation of improved stove programs in Guatemala: final report of project case studies. ESMAP Technical Paper No. 60. Washington DC: World Bank, pages 45-68. Available: http://www.esmap.org/sites/esmap.org/files/06004GuatemalaFinalEnglishforWeb.pdf [accessed 20 November 2013].

World Bank. 2004c. Case Study 3: Intervida project. In: Evaluation of improved stove programs in Guatemala: final report of project case studies. ESMAP Technical Paper No. 60. Washington DC: World Bank, pages 69-90. Available: http://www.esmap.org/sites/esmap.org/files/06004GuatemalaFinalEnglishforWeb.pdf [accessed 20 November 2013].

World Bank. 2010a. BCSIR Improved Cookstove Program, Phase II. In: Improved cookstoves and better health in Bangladesh: Lessons from household energy and sanitation programs. Washington DC: World Bank, pages 15-21. Available: http://www.hedconsulting.com/wordpress/wp-content/uploads/2012/02/improved_cookstoves_better_health.pdf [accessed 20 November 2013].

World Bank. 2010b. GTZ Sustainable Energy for Development Program: Improved Cookstoves Component. In: Improved cookstoves and better health in Bangladesh: Lessons from household energy and sanitation programs. Washington DC: World Bank, pages 21-24. Available: http://www.hedconsulting.com/wordpress/wp-content/uploads/2012/02/improved_cookstoves_better_health.pdf [accessed 20 November 2013].

World Bank. 2010c. USAID Reduction of Exposure to Indoor Air Pollution through Household Energy and Behavioral Improvement. In: Improved cookstoves and better health in Bangladesh: Lessons from household energy and sanitation programs. Washington DC: World Bank, pages 24-26. Available: http://www.hedconsulting.com/wordpress/wp-content/uploads/2012/02/improved_cookstoves_better_health.pdf [accessed 20 November
